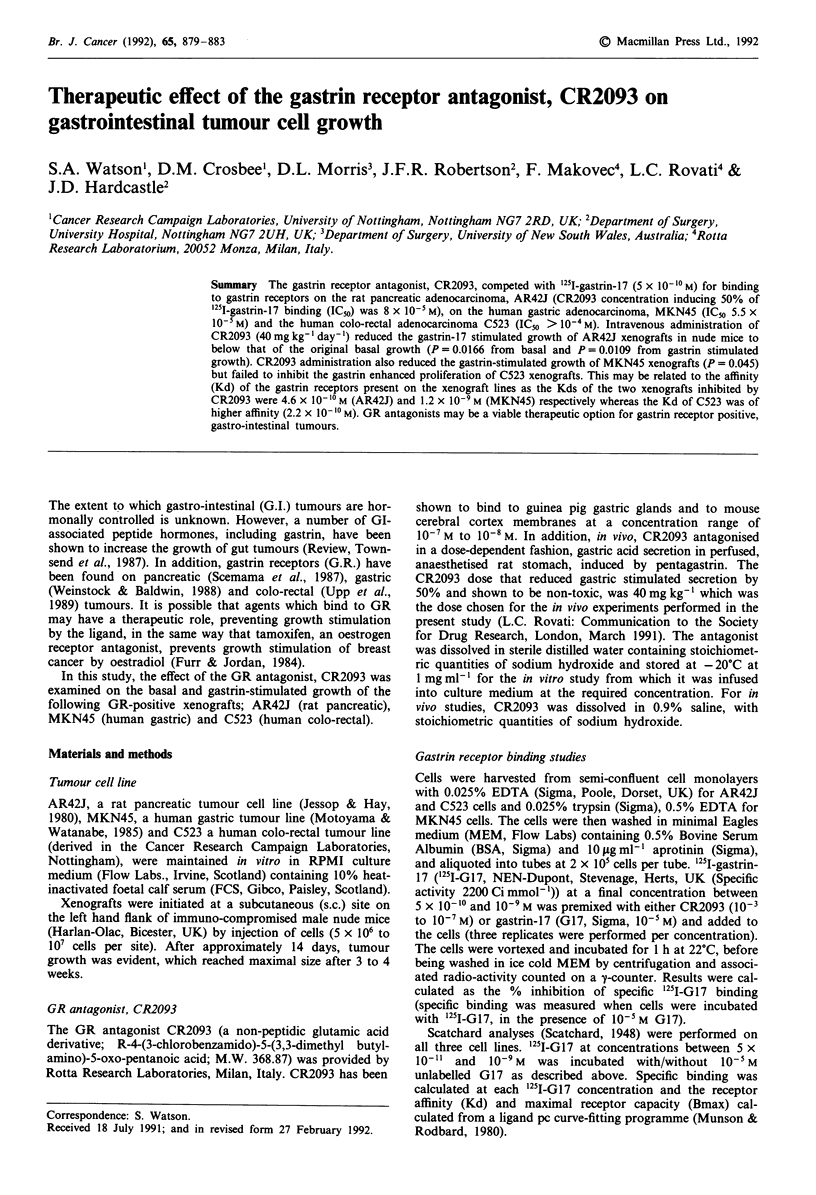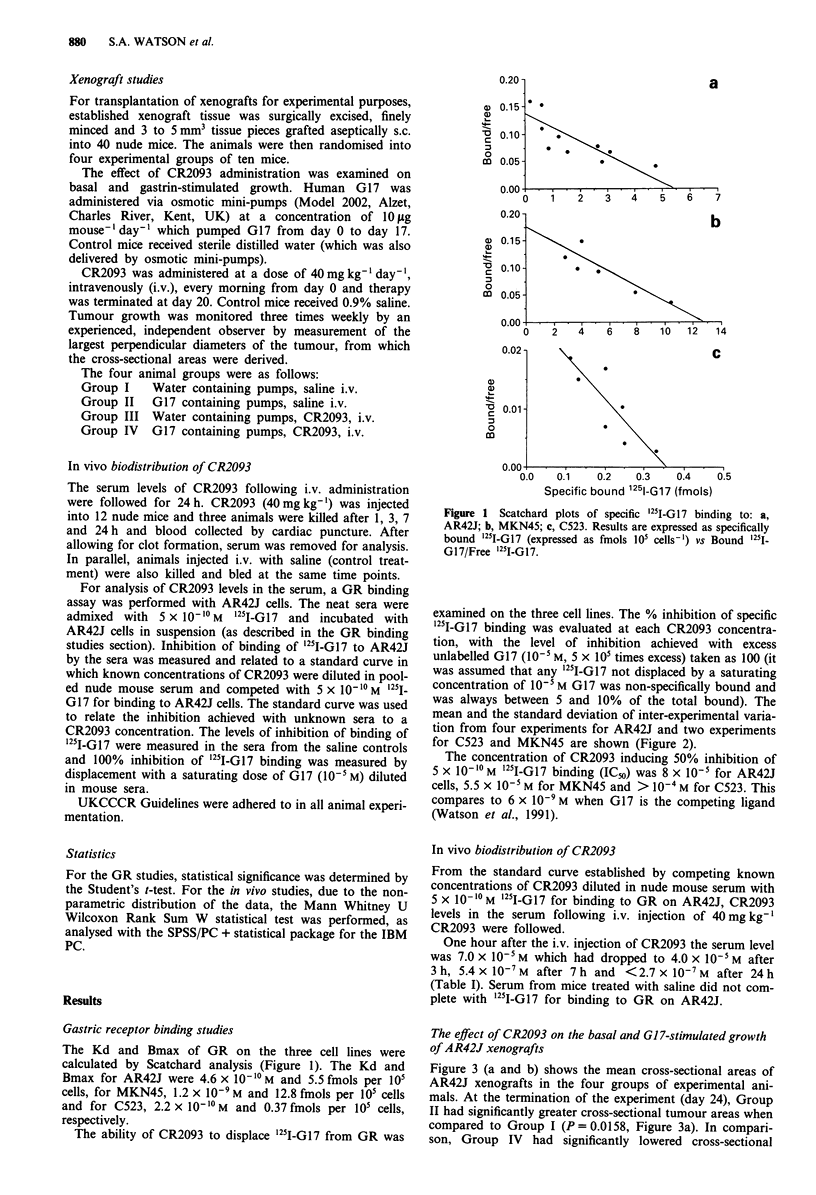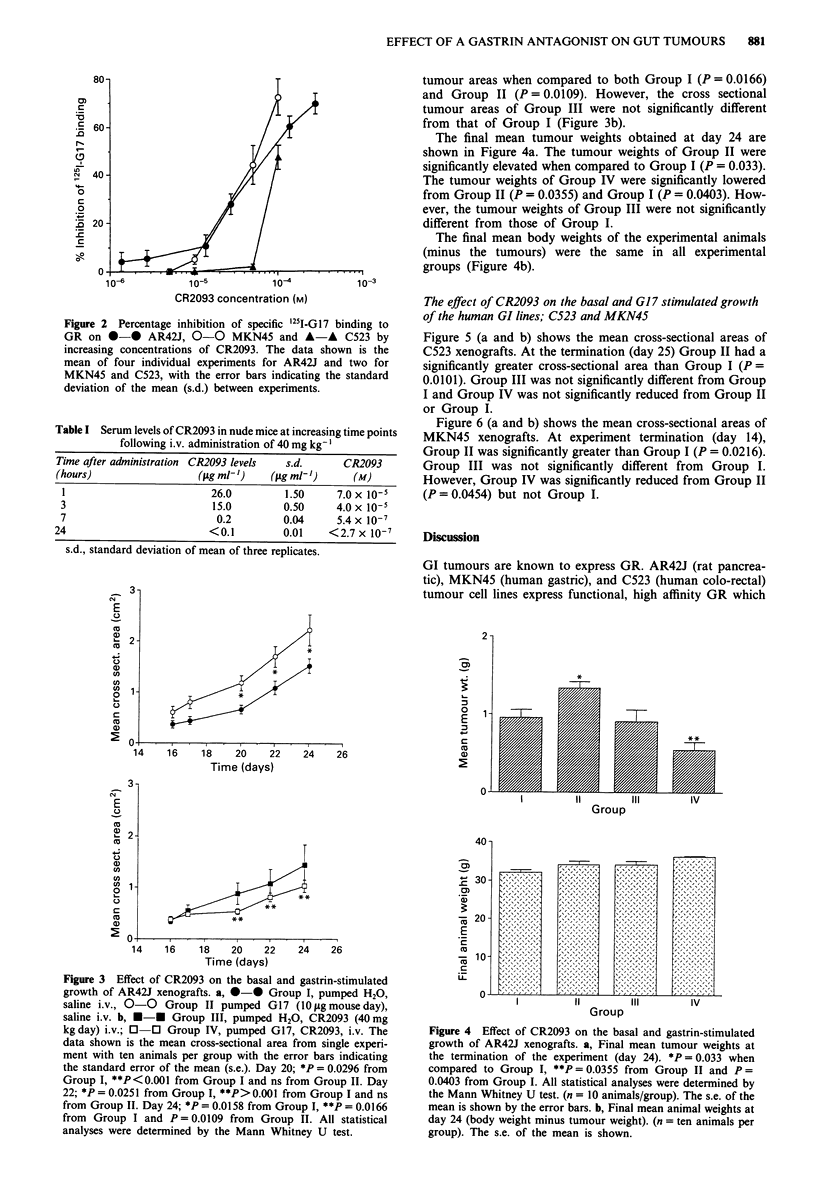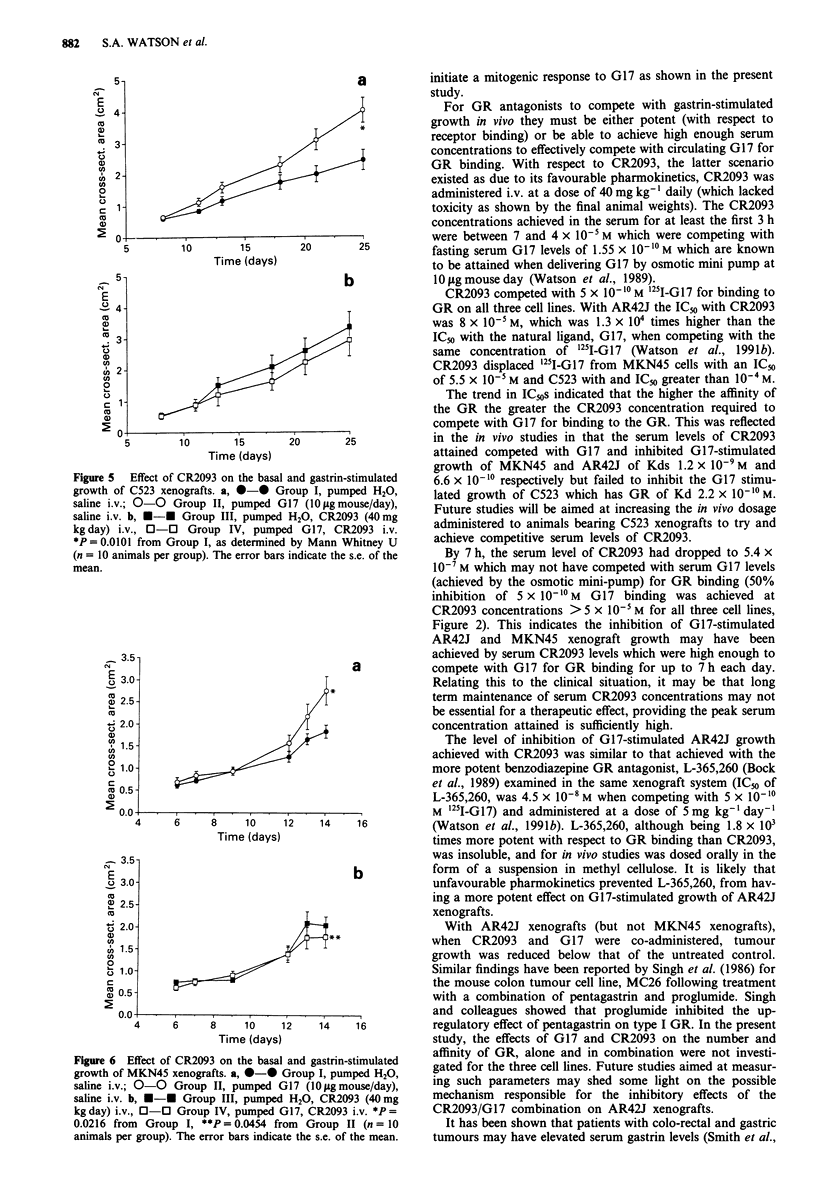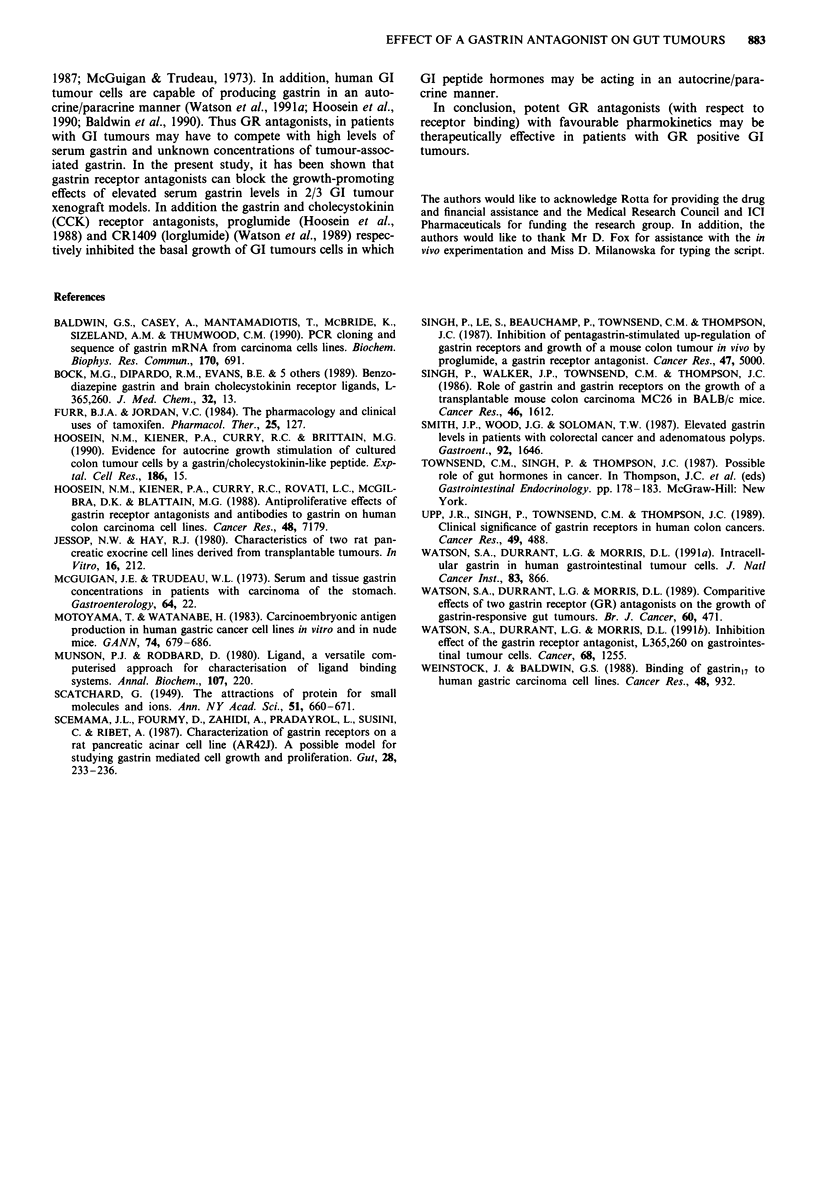# Therapeutic effect of the gastrin receptor antagonist, CR2093 on gastrointestinal tumour cell growth.

**DOI:** 10.1038/bjc.1992.184

**Published:** 1992-06

**Authors:** S. A. Watson, D. M. Crosbee, D. L. Morris, J. F. Robertson, F. Makovec, L. C. Rovati, J. D. Hardcastle

**Affiliations:** Cancer Research Campaign Laboratories, University of Nottingham, UK.

## Abstract

The gastrin receptor antagonist, CR2093, competed with 125I-gastrin-17 (5 x 10(-10) M) for binding to gastrin receptors on the rat pancreatic adenocarcinoma, AR42J (CR2093 concentration inducing 50% of 125I-gastrin-17 binding (IC50) was 8 x 10(-5) M), on the human gastric adenocarcinoma, MKN45 (IC50 5.5 x 10(-5) M) and the human colo-rectal adenocarcinoma C523 (IC50 greater than 10(-4) M). Intravenous administration of CR2093 (40 mg kg-1 day-1) reduced the gastrin-17 stimulated growth of AR42J xenografts in nude mice to below that of the original basal growth (P = 0.0166 from basal and P = 0.0109 from gastrin stimulated growth). CR2093 administration also reduced the gastrin-stimulated growth of MKN45 xenografts (P = 0.045) but failed to inhibit the gastrin enhanced proliferation of C523 xenografts. This may be related to the affinity (Kd) of the gastrin receptors present on the xenograft lines as the Kds of the two xenografts inhibited by CR2093 were 4.6 x 10(-10) M (AR42J) and 1.2 x 10(-9) M (MKN45) respectively whereas the Kd of C523 was of higher affinity (2.2 x 10(-10) M). GR antagonists may be a viable therapeutic option for gastrin receptor positive, gastro-intestinal tumours.


Br. J. Cancer (1992), 65, 879-883 © Macmillan Press Ltd., 1992

### Therapeutic effect of the gastrin receptor antagonist, CR2093 on gastrointestinal tumour cell growth

S.A. Watson[1], D.M. Crosbee[1], D.L. Morris[3], J.F.R. Robertson[2], F. Makovec[4], L.C. Rovati[4] & J.D. Hardcastle[2]

[1]*Cancer Research Campaign Laboratories, University of Nottingham, Nottingham NG7 2RD, UK;* [2]*Department of Surgery, University Hospital, Nottingham NG7 2UH, UK;* [3]*Department of Surgery, University of New South Wales, Australia;* [4]*Rotta Research Laboratorium, 20052 Monza, Milan, Italy.*

**Summary** The gastrin receptor antagonist, CR2093, competed with $^{125}$I-gastrin-17 ($5 \times 10^{-10}$ M) for binding to gastrin receptors on the rat pancreatic adenocarcinoma, AR42J (CR2093 concentration inducing 50% of $^{125}$I-gastrin-17 binding ($IC_{50}$) was $8 \times 10^{-5}$ M), on the human gastric adenocarcinoma, MKN45 ($IC_{50}$ $5.5 \times 10^{-5}$ M) and the human colo-rectal adenocarcinoma C523 ($IC_{50}$ $>10^{-4}$ M). Intravenous administration of CR2093 (40 mg kg$^{-1}$ day$^{-1}$) reduced the gastrin-17 stimulated growth of AR42J xenografts in nude mice to below that of the original basal growth ($P = 0.0166$ from basal and $P = 0.0109$ from gastrin stimulated growth). CR2093 administration also reduced the gastrin-stimulated growth of MKN45 xenografts ($P = 0.045$) but failed to inhibit the gastrin enhanced proliferation of C523 xenografts. This may be related to the affinity (Kd) of the gastrin receptors present on the xenograft lines as the Kds of the two xenografts inhibited by CR2093 were $4.6 \times 10^{-10}$ M (AR42J) and $1.2 \times 10^{-9}$ M (MKN45) respectively whereas the Kd of C523 was of higher affinity ($2.2 \times 10^{-10}$ M). GR antagonists may be a viable therapeutic option for gastrin receptor positive, gastro-intestinal tumours.

The extent to which gastro-intestinal (G.I.) tumours are hormonally controlled is unknown. However, a number of GI-associated peptide hormones, including gastrin, have been shown to increase the growth of gut tumours (Review, Townsend *et al.*, 1987). In addition, gastrin receptors (G.R.) have been found on pancreatic (Scemama *et al.*, 1987), gastric (Weinstock & Baldwin, 1988) and colo-rectal (Upp *et al.*, 1989) tumours. It is possible that agents which bind to GR may have a therapeutic role, preventing growth stimulation by the ligand, in the same way that tamoxifen, an oestrogen receptor antagonist, prevents growth stimulation of breast cancer by oestradiol (Furr & Jordan, 1984).

In this study, the effect of the GR antagonist, CR2093 was examined on the basal and gastrin-stimulated growth of the following GR-positive xenografts; AR42J (rat pancreatic), MKN45 (human gastric) and C523 (human colo-rectal).

### Materials and methods

### *Tumour cell line*

AR42J, a rat pancreatic tumour cell line (Jessop & Hay, 1980), MKN45, a human gastric tumour line (Motoyama & Watanabe, 1985) and C523 a human colo-rectal tumour line (derived in the Cancer Research Campaign Laboratories, Nottingham), were maintained *in vitro* in RPMI culture medium (Flow Labs., Irvine, Scotland) containing 10% heat-inactivated foetal calf serum (FCS, Gibco, Paisley, Scotland).

Xenografts were initiated at a subcutaneous (s.c.) site on the left hand flank of immuno-compromised male nude mice (Harlan-Olac, Bicester, UK) by injection of cells ($5 \times 10^6$ to $10^7$ cells per site). After approximately 14 days, tumour growth was evident, which reached maximal size after 3 to 4 weeks.

### *GR antagonist, CR2093*

The GR antagonist CR2093 (a non-peptidic glutamic acid derivative; R-4-(3-chlorobenzamido)-5-(3,3-dimethyl butyl-amino)-5-oxo-pentanoic acid; M.W. 368.87) was provided by Rotta Research Laboratories, Milan, Italy. CR2093 has been shown to bind to guinea pig gastric glands and to mouse cerebral cortex membranes at a concentration range of $10^{-7}$ M to $10^{-8}$ M. In addition, *in vivo*, CR2093 antagonised in a dose-dependent fashion, gastric acid secretion in perfused, anaesthetised rat stomach, induced by pentagastrin. The CR2093 dose that reduced gastric stimulated secretion by 50% and shown to be non-toxic, was 40 mg kg$^{-1}$ which was the dose chosen for the *in vivo* experiments performed in the present study (L.C. Rovati: Communication to the Society for Drug Research, London, March 1991). The antagonist was dissolved in sterile distilled water containing stoichiometric quantities of sodium hydroxide and stored at $-20$°C at 1 mg ml$^{-1}$ for the *in vitro* study from which it was infused into culture medium at the required concentration. For *in vivo* studies, CR2093 was dissolved in 0.9% saline, with stoichiometric quantities of sodium hydroxide.

### *Gastrin receptor binding studies*

Cells were harvested from semi-confluent cell monolayers with 0.025% EDTA (Sigma, Poole, Dorset, UK) for AR42J and C523 cells and 0.025% trypsin (Sigma), 0.5% EDTA for MKN45 cells. The cells were then washed in minimal Eagles medium (MEM, Flow Labs) containing 0.5% Bovine Serum Albumin (BSA, Sigma) and 10 $\mu$g ml$^{-1}$ aprotinin (Sigma), and aliquoted into tubes at $2 \times 10^5$ cells per tube. $^{125}$I-gastrin-17 ($^{125}$I-G17, NEN-Dupont, Stevenage, Herts, UK (Specific activity 2200 Ci mmol$^{-1}$)) at a final concentration between $5 \times 10^{-10}$ and $10^{-9}$ M was premixed with either CR2093 ($10^{-3}$ to $10^{-7}$ M) or gastrin-17 (G17, Sigma, $10^{-5}$ M) and added to the cells (three replicates were performed per concentration). The cells were vortexed and incubated for 1 h at 22°C, before being washed in ice cold MEM by centrifugation and associated radio-activity counted on a $\gamma$-counter. Results were calculated as the % inhibition of specific $^{125}$I-G17 binding (specific binding was measured when cells were incubated with $^{125}$I-G17, in the presence of $10^{-5}$ M G17).

Scatchard analyses (Scatchard, 1948) were performed on all three cell lines. $^{125}$I-G17 at concentrations between $5 \times 10^{-11}$ and $10^{-9}$ M was incubated with/without $10^{-5}$ M unlabelled G17 as described above. Specific binding was calculated at each $^{125}$I-G17 concentration and the receptor affinity (Kd) and maximal receptor capacity (Bmax) calculated from a ligand pc curve-fitting programme (Munson & Rodbard, 1980).

Correspondence: S. Watson.
Received 18 July 1991; and in revised form 27 February 1992.

*Xenograft studies*

For transplantation of xenografts for experimental purposes, established xenograft tissue was surgically excised, finely minced and 3 to 5 mm$^3$ tissue pieces grafted aseptically s.c. into 40 nude mice. The animals were then randomised into four experimental groups of ten mice.

The effect of CR2093 administration was examined on basal and gastrin-stimulated growth. Human G17 was administered via osmotic mini-pumps (Model 2002, Alzet, Charles River, Kent, UK) at a concentration of 10 $\mu$g mouse$^{-1}$ day$^{-1}$ which pumped G17 from day 0 to day 17. Control mice received sterile distilled water (which was also delivered by osmotic mini-pumps).

CR2093 was administered at a dose of 40 mg kg$^{-1}$ day$^{-1}$, intravenously (i.v.), every morning from day 0 and therapy was terminated at day 20. Control mice received 0.9% saline. Tumour growth was monitored three times weekly by an experienced, independent observer by measurement of the largest perpendicular diameters of the tumour, from which the cross-sectional areas were derived.

The four animal groups were as follows:

Group I Water containing pumps, saline i.v.
Group II G17 containing pumps, saline i.v.
Group III Water containing pumps, CR2093, i.v.
Group IV G17 containing pumps, CR2093, i.v.

*In vivo biodistribution of CR2093*

The serum levels of CR2093 following i.v. administration were followed for 24 h. CR2093 (40 mg kg$^{-1}$) was injected into 12 nude mice and three animals were killed after 1, 3, 7 and 24 h and blood collected by cardiac puncture. After allowing for clot formation, serum was removed for analysis. In parallel, animals injected i.v. with saline (control treatment) were also killed and bled at the same time points.

For analysis of CR2093 levels in the serum, a GR binding assay was performed with AR42J cells. The neat sera were admixed with $5 \times 10^{-10}$ M $^{125}$I-G17 and incubated with AR42J cells in suspension (as described in the GR binding studies section). Inhibition of binding of $^{125}$I-G17 to AR42J by the sera was measured and related to a standard curve in which known concentrations of CR2093 were diluted in pooled nude mouse serum and competed with $5 \times 10^{-10}$ M $^{125}$I-G17 for binding to AR42J cells. The standard curve was used to relate the inhibition achieved with unknown sera to a CR2093 concentration. The levels of inhibition of binding of $^{125}$I-G17 were measured in the sera from the saline controls and 100% inhibition of $^{125}$I-G17 binding was measured by displacement with a saturating dose of G17 ($10^{-5}$ M) diluted in mouse sera.

UKCCCR Guidelines were adhered to in all animal experimentation.

*Statistics*

For the GR studies, statistical significance was determined by the Student's *t*-test. For the *in vivo* studies, due to the non-parametric distribution of the data, the Mann Whitney U Wilcoxon Rank Sum W statistical test was performed, as analysed with the SPSS/PC + statistical package for the IBM PC.

### Results

*Gastric receptor binding studies*

The Kd and Bmax of GR on the three cell lines were calculated by Scatchard analysis (Figure 1). The Kd and Bmax for AR42J were $4.6 \times 10^{-10}$ M and 5.5 fmols per 10$^5$ cells, for MKN45, $1.2 \times 10^{-9}$ M and 12.8 fmols per 10$^5$ cells and for C523, $2.2 \times 10^{-10}$ M and 0.37 fmols per 10$^5$ cells, respectively.

The ability of CR2093 to displace $^{125}$I-G17 from GR was

**Figure 1** Scatchard plots of specific $^{125}$I-G17 binding to: **a**, AR42J; **b**, MKN45; **c**, C523. Results are expressed as specifically bound $^{125}$I-G17 (expressed as fmols 10$^5$ cells$^{-1}$) *vs* Bound $^{125}$I-G17/Free $^{125}$I-G17.

examined on the three cell lines. The % inhibition of specific $^{125}$I-G17 binding was evaluated at each CR2093 concentration, with the level of inhibition achieved with excess unlabelled G17 ($10^{-5}$ M, $5 \times 10^5$ times excess) taken as 100 (it was assumed that any $^{125}$I-G17 not displaced by a saturating concentration of $10^{-5}$ M G17 was non-specifically bound and was always between 5 and 10% of the total bound). The mean and the standard deviation of inter-experimental variation from four experiments for AR42J and two experiments for C523 and MKN45 are shown (Figure 2).

The concentration of CR2093 inducing 50% inhibition of $5 \times 10^{-10}$ M $^{125}$I-G17 binding (IC$_{50}$) was $8 \times 10^{-5}$ for AR42J cells, $5.5 \times 10^{-5}$ M for MKN45 and $> 10^{-4}$ M for C523. This compares to $6 \times 10^{-9}$ M when G17 is the competing ligand (Watson *et al.*, 1991).

*In vivo biodistribution of CR2093*

From the standard curve established by competing known concentrations of CR2093 diluted in nude mouse serum with $5 \times 10^{-10}$ M $^{125}$I-G17 for binding to GR on AR42J, CR2093 levels in the serum following i.v. injection of 40 mg kg$^{-1}$ CR2093 were followed.

One hour after the i.v. injection of CR2093 the serum level was $7.0 \times 10^{-5}$ M which had dropped to $4.0 \times 10^{-5}$ M after 3 h, $5.4 \times 10^{-7}$ M after 7 h and $< 2.7 \times 10^{-7}$ M after 24 h (Table I). Serum from mice treated with saline did not complete with $^{125}$I-G17 for binding to GR on AR42J.

*The effect of CR2093 on the basal and G17-stimulated growth of AR42J xenografts*

Figure 3 (a and b) shows the mean cross-sectional areas of AR42J xenografts in the four groups of experimental animals. At the termination of the experiment (day 24), Group II had significantly greater cross-sectional tumour areas when compared to Group I ($P = 0.0158$, Figure 3a). In comparison, Group IV had significantly lowered cross-sectional

**Figure 2** Percentage inhibition of specific $^{125}$I-G17 binding to GR on ●—● AR42J, ○—○ MKN45 and ▲—▲ C523 by increasing concentrations of CR2093. The data shown is the mean of four individual experiments for AR42J and two for MKN45 and C523, with the error bars indicating the standard deviation of the mean (s.d.) between experiments.

**Table I** Serum levels of CR2093 in nude mice at increasing time points following i.v. administration of 40 mg $kg^{-1}$

| *Time after administration (hours)* | *CR2093 levels ($\mu g\ ml^{-1}$)* | *s.d. ($\mu g\ ml^{-1}$)* | *CR2093 (M)* |
|---|---|---|---|
| 1 | 26.0 | 1.50 | $7.0 \times 10^{-5}$ |
| 3 | 15.0 | 0.50 | $4.0 \times 10^{-5}$ |
| 7 | 0.2 | 0.04 | $5.4 \times 10^{-7}$ |
| 24 | <0.1 | 0.01 | $<2.7 \times 10^{-7}$ |

s.d., standard deviation of mean of three replicates.

**Figure 3** Effect of CR2093 on the basal and gastrin-stimulated growth of AR42J xenografts. **a**, ●—● Group I, pumped $H_2O$, saline i.v., ○—○ Group II pumped G17 (10 µg mouse day), saline i.v. **b**, ■—■ Group III, pumped $H_2O$, CR2093 (40 mg kg day) i.v.; □—□ Group IV, pumped G17, CR2093, i.v. The data shown is the mean cross-sectional area from single experiment with ten animals per group with the error bars indicating the standard error of the mean (s.e.). Day 20; *$P = 0.0296$ from Group I, **$P < 0.001$ from Group I and ns from Group II. Day 22; *$P = 0.0251$ from Group I, **$P > 0.001$ from Group I and ns from Group II. Day 24; *$P = 0.0158$ from Group I, **$P = 0.0166$ from Group I and $P = 0.0109$ from Group II. All statistical analyses were determined by the Mann Whitney U test.

tumour areas when compared to both Group I ($P = 0.0166$) and Group II ($P = 0.0109$). However, the cross sectional tumour areas of Group III were not significantly different from that of Group I (Figure 3b).

The final mean tumour weights obtained at day 24 are shown in Figure 4a. The tumour weights of Group II were significantly elevated when compared to Group I ($P = 0.033$). The tumour weights of Group IV were significantly lowered from Group II ($P = 0.0355$) and Group I ($P = 0.0403$). However, the tumour weights of Group III were not significantly different from those of Group I.

The final mean body weights of the experimental animals (minus the tumours) were the same in all experimental groups (Figure 4b).

### *The effect of CR2093 on the basal and G17 stimulated growth of the human GI lines; C523 and MKN45*

Figure 5 (a and b) shows the mean cross-sectional areas of C523 xenografts. At the termination (day 25) Group II had a significantly greater cross-sectional area than Group I ($P = 0.0101$). Group III was not significantly different from Group I and Group IV was not significantly reduced from Group II or Group I.

Figure 6 (a and b) shows the mean cross-sectional areas of MKN45 xenografts. At experiment termination (day 14), Group II was significantly greater than Group I ($P = 0.0216$). Group III was not significantly different from Group I. However, Group IV was significantly reduced from Group II ($P = 0.0454$) but not Group I.

### Discussion

GI tumours are known to express GR. AR42J (rat pancreatic), MKN45 (human gastric), and C523 (human colo-rectal) tumour cell lines express functional, high affinity GR which

**Figure 4** Effect of CR2093 on the basal and gastrin-stimulated growth of AR42J xenografts. **a**, Final mean tumour weights at the termination of the experiment (day 24). *$P = 0.033$ when compared to Group I, **$P = 0.0355$ from Group II and $P = 0.0403$ from Group I. All statistical analyses were determined by the Mann Whitney U test. ($n = 10$ animals/group). The s.e. of the mean is shown by the error bars. **b**, Final mean animal weights at day 24 (body weight minus tumour weight). ($n$ = ten animals per group). The s.e. of the mean is shown.

**Figure 5** Effect of CR2093 on the basal and gastrin-stimulated growth of C523 xenografts. **a**, ●—● Group I, pumped $H_2O$, saline i.v.; O—O Group II, pumped G17 (10 μg mouse/day), saline i.v. **b**, ■—■ Group III, pumped $H_2O$, CR2093 (40 mg kg day) i.v., □—□ Group IV, pumped G17, CR2093 i.v. *$P = 0.0101$ from Group I, as determined by Mann Whitney U ($n = 10$ animals per group). The error bars indicate the s.e. of the mean.

**Figure 6** Effect of CR2093 on the basal and gastrin-stimulated growth of MKN45 xenografts. **a**, ●—● Group I, pumped $H_2O$, saline i.v.; O—O Group II, pumped G17 (10 μg mouse/day), saline i.v. **b**, ■—■ Group III, pumped $H_2O$, CR2093 (40 mg kg day) i.v., □—□ Group IV, pumped G17, CR2093 i.v. *$P = 0.0216$ from Group I, **$P = 0.0454$ from Group II ($n = 10$ animals per group). The error bars indicate the s.e. of the mean.

initiate a mitogenic response to G17 as shown in the present study.

For GR antagonists to compete with gastrin-stimulated growth *in vivo* they must be either potent (with respect to receptor binding) or be able to achieve high enough serum concentrations to effectively compete with circulating G17 for GR binding. With respect to CR2093, the latter scenario existed as due to its favourable pharmokinetics, CR2093 was administered i.v. at a dose of 40 mg $kg^{-1}$ daily (which lacked toxicity as shown by the final animal weights). The CR2093 concentrations achieved in the serum for at least the first 3 h were between 7 and $4 \times 10^{-5}$ M which were competing with fasting serum G17 levels of $1.55 \times 10^{-10}$ M which are known to be attained when delivering G17 by osmotic mini pump at 10 μg mouse day (Watson *et al.*, 1989).

CR2093 competed with $5 \times 10^{-10}$ M $^{125}$I-G17 for binding to GR on all three cell lines. With AR42J the $IC_{50}$ with CR2093 was $8 \times 10^{-5}$ M, which was $1.3 \times 10^4$ times higher than the $IC_{50}$ with the natural ligand, G17, when competing with the same concentration of $^{125}$I-G17 (Watson *et al.*, 1991*b*). CR2093 displaced $^{125}$I-G17 from MKN45 cells with an $IC_{50}$ of $5.5 \times 10^{-5}$ M and C523 with and $IC_{50}$ greater than $10^{-4}$ M.

The trend in $IC_{50}$s indicated that the higher the affinity of the GR the greater the CR2093 concentration required to compete with G17 for binding to the GR. This was reflected in the *in vivo* studies in that the serum levels of CR2093 attained competed with G17 and inhibited G17-stimulated growth of MKN45 and AR42J of Kds $1.2 \times 10^{-9}$ M and $6.6 \times 10^{-10}$ respectively but failed to inhibit the G17 stimulated growth of C523 which has GR of Kd $2.2 \times 10^{-10}$ M. Future studies will be aimed at increasing the *in vivo* dosage administered to animals bearing C523 xenografts to try and achieve competitive serum levels of CR2093.

By 7 h, the serum level of CR2093 had dropped to $5.4 \times 10^{-7}$ M which may not have competed with serum G17 levels (achieved by the osmotic mini-pump) for GR binding (50% inhibition of $5 \times 10^{-10}$ M G17 binding was achieved at CR2093 concentrations $> 5 \times 10^{-5}$ M for all three cell lines, Figure 2). This indicates the inhibition of G17-stimulated AR42J and MKN45 xenograft growth may have been achieved by serum CR2093 levels which were high enough to compete with G17 for GR binding for up to 7 h each day. Relating this to the clinical situation, it may be that long term maintenance of serum CR2093 concentrations may not be essential for a therapeutic effect, providing the peak serum concentration attained is sufficiently high.

The level of inhibition of G17-stimulated AR42J growth achieved with CR2093 was similar to that achieved with the more potent benzodiazepine GR antagonist, L-365,260 (Bock *et al.*, 1989) examined in the same xenograft system ($IC_{50}$ of L-365,260, was $4.5 \times 10^{-8}$ M when competing with $5 \times 10^{-10}$ M $^{125}$I-G17) and administered at a dose of 5 mg $kg^{-1}$ $day^{-1}$ (Watson *et al.*, 1991*b*). L-365,260, although being $1.8 \times 10^3$ times more potent with respect to GR binding than CR2093, was insoluble, and for *in vivo* studies was dosed orally in the form of a suspension in methyl cellulose. It is likely that unfavourable pharmokinetics prevented L-365,260, from having a more potent effect on G17-stimulated growth of AR42J xenografts.

With AR42J xenografts (but not MKN45 xenografts), when CR2093 and G17 were co-administered, tumour growth was reduced below that of the untreated control. Similar findings have been reported by Singh *et al.* (1986) for the mouse colon tumour cell line, MC26 following treatment with a combination of pentagastrin and proglumide. Singh and colleagues showed that proglumide inhibited the up-regulatory effect of pentagastrin on type I GR. In the present study, the effects of G17 and CR2093 on the number and affinity of GR, alone and in combination were not investigated for the three cell lines. Future studies aimed at measuring such parameters may shed some light on the possible mechanism responsible for the inhibitory effects of the CR2093/G17 combination on AR42J xenografts.

It has been shown that patients with colo-rectal and gastric tumours may have elevated serum gastrin levels (Smith *et al.*,

1987; McGuigan & Trudeau, 1973). In addition, human GI tumour cells are capable of producing gastrin in an autocrine/paracrine manner (Watson *et al.*, 1991*a*; Hoosein *et al.*, 1990; Baldwin *et al.*, 1990). Thus GR antagonists, in patients with GI tumours may have to compete with high levels of serum gastrin and unknown concentrations of tumour-associated gastrin. In the present study, it has been shown that gastrin receptor antagonists can block the growth-promoting effects of elevated serum gastrin levels in 2/3 GI tumour xenograft models. In addition the gastrin and cholecystokinin (CCK) receptor antagonists, proglumide (Hoosein *et al.*, 1988) and CR1409 (lorglumide) (Watson *et al.*, 1989) respectively inhibited the basal growth of GI tumours cells in which GI peptide hormones may be acting in an autocrine/paracrine manner.

In conclusion, potent GR antagonists (with respect to receptor binding) with favourable pharmokinetics may be therapeutically effective in patients with GR positive GI tumours.

The authors would like to acknowledge Rotta for providing the drug and financial assistance and the Medical Research Council and ICI Pharmaceuticals for funding the research group. In addition, the authors would like to thank Mr D. Fox for assistance with the *in vivo* experimentation and Miss D. Milanowska for typing the script.

### References

BALDWIN, G.S., CASEY, A., MANTAMADIOTIS, T., MCBRIDE, K., SIZELAND, A.M. & THUMWOOD, C.M. (1990). PCR cloning and sequence of gastrin mRNA from carcinoma cells lines. *Biochem. Biophys. Res. Commun.*, **170**, 691.

BOCK, M.G., DIPARDO, R.M., EVANS, B.E. & 5 others (1989). Benzodiazepine gastrin and brain cholecystokinin receptor ligands, L-365,260. *J. Med. Chem.*, **32**, 13.

FURR, B.J.A. & JORDAN, V.C. (1984). The pharmacology and clinical uses of tamoxifen. *Pharmacol. Ther.*, **25**, 127.

HOOSEIN, N.M., KIENER, P.A., CURRY, R.C. & BRITTAIN, M.G. (1990). Evidence for autocrine growth stimulation of cultured colon tumour cells by a gastrin/cholecystokinin-like peptide. *Exptal. Cell Res.*, **186**, 15.

HOOSEIN, N.M., KIENER, P.A., CURRY, R.C., ROVATI, L.C., MCGILBRA, D.K. & BLATTAIN, M.G. (1988). Antiproliferative effects of gastrin receptor antagonists and antibodies to gastrin on human colon carcinoma cell lines. *Cancer Res.*, **48**, 7179.

JESSOP, N.W. & HAY, R.J. (1980). Characteristics of two rat pancreatic exocrine cell lines derived from transplantable tumours. *In Vitro*, **16**, 212.

MCGUIGAN, J.E. & TRUDEAU, W.L. (1973). Serum and tissue gastrin concentrations in patients with carcinoma of the stomach. *Gastroenterology*, **64**, 22.

MOTOYAMA, T. & WATANABE, H. (1983). Carcinoembryonic antigen production in human gastric cancer cell lines *in vitro* and in nude mice. *GANN*, **74**, 679-686.

MUNSON, P.J. & RODBARD, D. (1980). Ligand, a versatile computerised approach for characterisation of ligand binding systems. *Annal. Biochem.*, **107**, 220.

SCATCHARD, G. (1949). The attractions of protein for small molecules and ions. *Ann. NY Acad. Sci.*, **51**, 660-671.

SCEMAMA, J.L., FOURMY, D., ZAHIDI, A., PRADAYROL, L., SUSINI, C. & RIBET, A. (1987). Characterization of gastrin receptors on a rat pancreatic acinar cell line (AR42J). A possible model for studying gastrin mediated cell growth and proliferation. *Gut*, **28**, 233-236.

SINGH, P., LE, S., BEAUCHAMP, P., TOWNSEND, C.M. & THOMPSON, J.C. (1987). Inhibition of pentagastrin-stimulated up-regulation of gastrin receptors and growth of a mouse colon tumour *in vivo* by proglumide, a gastrin receptor antagonist. *Cancer Res.*, **47**, 5000.

SINGH, P., WALKER, J.P., TOWNSEND, C.M. & THOMPSON, J.C. (1986). Role of gastrin and gastrin receptors on the growth of a transplantable mouse colon carcinoma MC26 in BALB/c mice. *Cancer Res.*, **46**, 1612.

SMITH, J.P., WOOD, J.G. & SOLOMAN, T.W. (1987). Elevated gastrin levels in patients with colorectal cancer and adenomatous polyps. *Gastroent.*, **92**, 1646.

TOWNSEND, C.M., SINGH, P. & THOMPSON, J.C. (1987). Possible role of gut hormones in cancer. In Thompson, J.C. *et al.* (eds) *Gastrointestinal Endocrinology*. pp. 178-183. McGraw-Hill: New York.

UPP, J.R., SINGH, P., TOWNSEND, C.M. & THOMPSON, J.C. (1989). Clinical significance of gastrin receptors in human colon cancers. *Cancer Res.*, **49**, 488.

WATSON, S.A., DURRANT, L.G. & MORRIS, D.L. (1991*a*). Intracellular gastrin in human gastrointestinal tumour cells. *J. Natl Cancer Inst.*, **83**, 866.

WATSON, S.A., DURRANT, L.G. & MORRIS, D.L. (1989). Comparitive effects of two gastrin receptor (GR) antagonists on the growth of gastrin-responsive gut tumours. *Br. J. Cancer*, **60**, 471.

WATSON, S.A., DURRANT, L.G. & MORRIS, D.L. (1991*b*). Inhibition effect of the gastrin receptor antagonist, L365,260 on gastrointestinal tumour cells. *Cancer*, **68**, 1255.

WEINSTOCK, J. & BALDWIN, G.S. (1988). Binding of gastrin$_{17}$ to human gastric carcinoma cell lines. *Cancer Res.*, **48**, 932.